# Urethral Duplication with a Cystic Phallic Urethra Associated with a Uterus Didelphys, Partial Agenesis of the Tibia, and an Equinovarus Foot

**DOI:** 10.1155/2018/3827820

**Published:** 2018-03-12

**Authors:** Edwige Kafando, Boniface Moifo, Landry Mbouche, Derek Ndangoh, Evelyn Mah, Faustin Mouafo Tambo

**Affiliations:** ^1^Faculty of Medicine and Biomedical Sciences, University of Yaounde I, Yaounde, Cameroon; ^2^Department of Radiology, Yaounde Gynaeco-Obstetric and Pediatric Hospital, Yaounde, Cameroon; ^3^Department of Pediatric Surgery, Yaounde Gynaeco-Obstetric and Pediatric Hospital, Yaounde, Cameroon; ^4^Department of Neonatology, Yaounde Gynaeco-Obstetric and Pediatric Hospital, Yaounde, Cameroon

## Abstract

Urethral duplication is a rare congenital malformation, especially in females. It may be associated with complex urogenital malformations, but the association with a cystic phallic urethra and a uterus didelphys is exceptional. We report a case of a newborn with urethral duplication, with the accessory urethra exteriorized by a large cyst, associated with a uterus didelphys and bone malformations. We discuss the clinical, radiographic, and therapeutic aspects as well as a literature review.

## 1. Introduction

Urethral duplications are among the rarest congenital malformations of the urinary tract, with about 500 cases described in the literature in 2008 [1]. They are defined by the existence of 2 urethras, with several reported anatomical variants, associated or not with genital malformations. They are encountered most often in males and are of exceptional occurrence in females [2]. To the best of our knowledge, no case having cystic phallic urethra with uterus didelphys has been described in literature. We, therefore, report clinical and radiological features of this case along with brief treatment.

## 2. Observation

**Figure 1:**
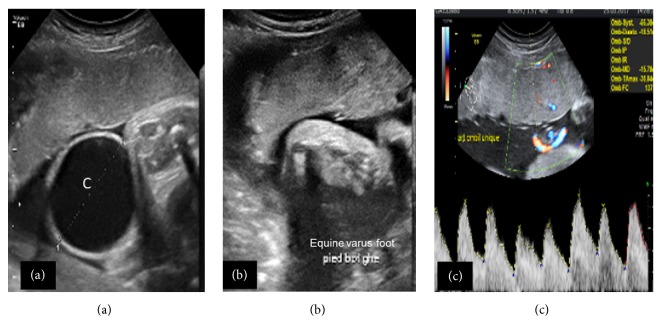
Morphological anomalies seen at antenatal ultrasound. (a) Cystic perineal mass (C), (b) equinovarus left foot, and (c) umbilical cord with a single artery.

**Figure 2:**
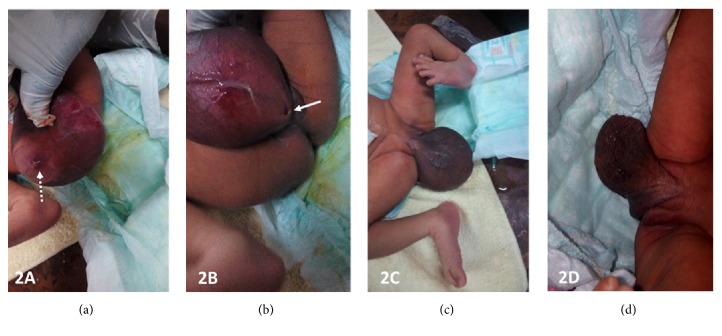
Postnatal photographs of the newborn on day 2 of life. (a) Cystic vulvar mass with an orifice at the anterior superior part of the mass (broken arrow) emitting urine intermittently. (b) Below the cyst was another orifice (full arrow) emitting urine more regularly. (c) Equinovarus left foot. (d) Emptying of the mass after voiding.

A 2-day-old female newborn was referred to us for malformation assessment. A perineal cyst, a left equinovarus foot, and a single umbilical artery were demonstrated during third trimester obstetric ultrasound (Figure 1). Her mother is a 17-year-old primiparous lady whose pregnancy had been well monitored and without any particular incident. The delivery was done by Caesarean section. Physical examination of the newborn confirmed the presence of a cystic mass attached to the vulva, with an orifice in the anterosuperior part that occasionally emitted urine, with the mass emptying and gradually filling with the urine (Figure 2). Below the cyst was another orifice, through which the urine flowed more regularly. The anus was normal.

**Figure 3:**
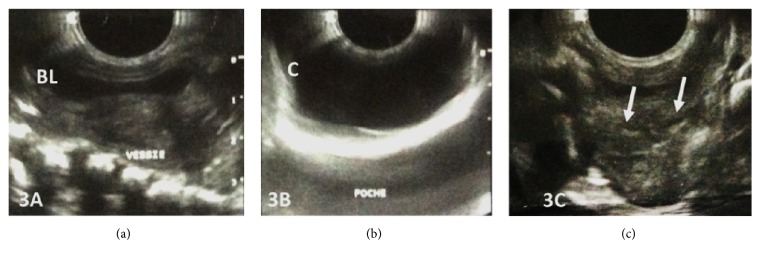
Postnatal pelvic and perineal ultrasound of the baby. (a-b) Suprapubic cross sections showing bladder (BL) and transperineal view showing cystic mass (C). (c) Uterus didelphys with the endometrial echogenic lines of the two hemiuteri (arrows).

On ultrasound, this vulvar mass presented with thick walls and an anechoic content. Examination of the urinary tract revealed normal kidneys and a normally located bladder, behind which two uterine hemimatrices suggestive of a uterus didelphys were visualized (Figure 3).

**Figure 4:**
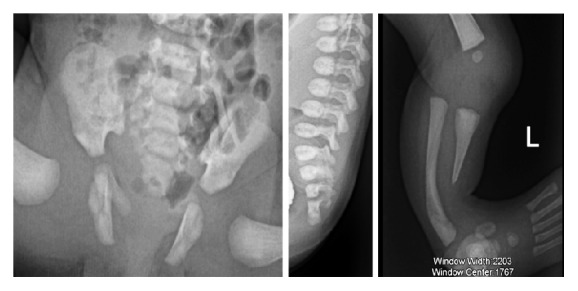
Radiographs centered on the anomalies of the bony structures (frontal view of the pelvis, lateral views of the lumbosacral spine and the left leg): VACTERL. Pubic diastasis, fusion of the last sacral vertebrae with agenesis of the coccyx, agenesis of the distal half of the tibia with fibular curvature, and an equinovarus deformation of the left foot.

**Figure 5:**
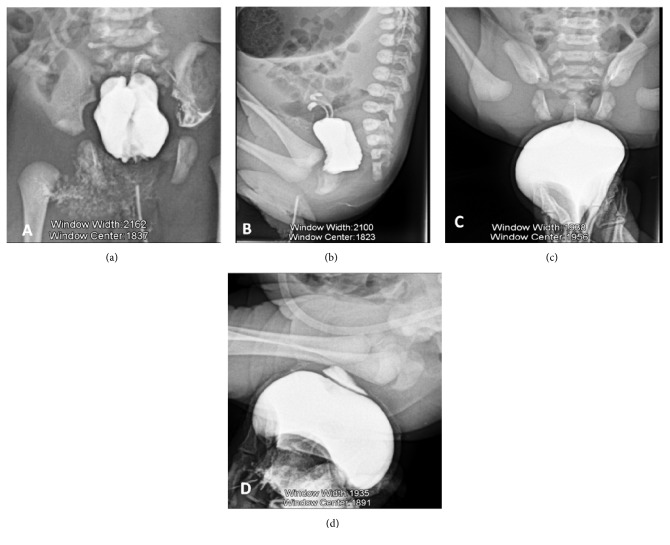
Opacification with iodine-based contrast media. (a) and (b) Through the orifice below the cyst: evidence of two hemiuteri and tubes suggestive of a uterus didelphys. (c) and (d) Through the anterosuperior orifice: opacification of the vulvar cyst and of the phallic accessory urethra with the onset of bladder filling on the lateral view.

In order to investigate the relationship between the cyst and the bladder, a retrograde urethrocystography was performed with simultaneous opacification of the two urine evacuation orifices. An X-ray of the left lower limb was performed to assess the deformity of the foot and leg. The analysis of the bony structures revealed a fusion of the last two sacral vertebrae with coccygeal agenesis and pubic diastasis and partial agenesis of the distal half of the left tibia with an equinovarus distortion of the foot (Figure 4). Opacification through the orifice below the cyst initially led to the filling of two oblong cavities, each continuous by a filamentous and tortuous structure suggestive of the tubal morphology, with the left being opacified right up to the distal portions and peritoneal spillage of the contrast medium, thus confirming the uterus didelphys (Figures 5(a) and 5(b)). During the evacuation of the contrast medium, there was reflux into the cystic mass with elimination through the two orifices, which made it possible to affirm the existence of a urogenital sinus thus explaining the communication between the urinary and genital tracts. The contrast medium injected through the orifice on the cyst completely molded the walls of the mass, which were regular and thin. Pressure maneuvers revealed a short urethral pathway to the bladder that was partially opacified (Figures 5(c) and 5(d)). With these images, we suggested a complex urogenital malformation associating a uterus didelphys and a urethral duplicity: the main urethra communicating with the vagina by the persistence of the urogenital sinus opening externally via the orifice below the cyst; the accessory phallic urethra opens externally to the vulva via a voluminous cyst with an orifice at its anterosuperior surface and emitting urine intermittently. A complementary pelvic MRI would have been more demonstrative but could not be performed due to lack of financial means.

**Figure 6:**
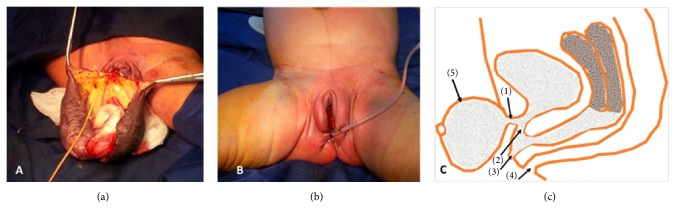
(a-b) Intraoperative view: resection of the cyst and phallic accessory urethra. (c) Illustrative diagram of urethral duplicity: (1) accessory phallic urethra, (2) main urethra, (3) urogenital sinus, (4) anus, and (5) clitoral cyst.

A multistep surgical management was proposed, the first consisting of an excision of the phallic accessory urethra with resection of the cyst and a vaginoplasty, thus allowing a complete separation of the urinary tract of the genital structures (Figures 6(a) and 6(b)): the repair of the malformations of the left lower limb having been deferred.

## 3. Discussion

Anatomically, urethral duplication is characterized by two urethras. One urethra is usually normal and the other is an accessory urethra. It may be either frontal or sagittal, and depending on whether the external orifice of the accessory urethra opens above or below the external orifice of the main urethra (the one with a markedly stronger urine flow), this distinguishes epispadic and hypospadic urethral duplications [1]. The male urethral duplication is divided into three types according to Effman's classification [3]. In girls, a classification of 5 types has been proposed [4]:

- Type I: double urethra and double bladder
- Type II: double urethra with single bladder
- Type III: accessory urethra posterior to the normal channel
- Type IV: double proximal urethra and single distal urethra
- Type V: single proximal urethra and duplicated distal urethra.

Other authors have described two varieties: a duplication in the horizontal plane with 2 parallel channels and a duplication in the sagittal plane, with a posterior functional main channel opening into the vagina and a phallic accessory channel [2, 5]. Our findings were suggestive of the latter and were confirmed during the surgery (Figure 6(c)).

Various theories have been proposed for development of urethral duplication, but no single theory explains all the various types of anomalies [1]. The most widely accepted theory for the cause of complete urethral duplication is that of Patten and Barry. According to them, an abnormal relationship exists between the lateral anlagen of the genital tubercle and the ventral end of the cloacal membrane. [1, 4, 6]. The opening of the urethra in the vagina corresponds to a defect of the urethrovaginal septum which may be total, allowing a direct communication of the bladder with the vagina, or more frequently partial [2].

**Figure 7:**
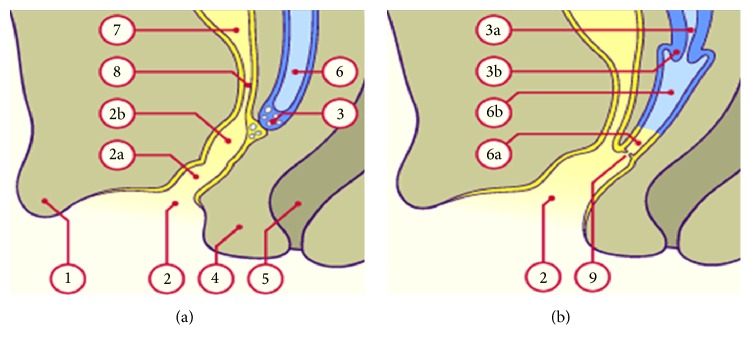
(a-b) Diagram of the urogenital sinus (UGS) at about 12 weeks and 9 months (source: http://www.embryology.ch/francais/ugenital/genitinterne06.html). (a) Urogenital sinus (UGS) at about 12 weeks: (1) genital tubercle, (2) vestibule, (2a) urogenital sinus (UGS) phallic portion, (2b) UGS pelvic portion, (3) vaginal epithelial blade, (4) perineum, (5) rectum, (6) uterovaginal canal, (7) bladder, and (8) urethra. (b) Definitive vestibule, uterus, and vagina at about the 9th month: (2) definitive vestibule, (3a) uterus (body), (3b) uterus (cervix), (6a) lower 1/4 of the vagina (endoblast), (6b) upper 3/4 of the vagina (mesoblast), and (9) hymen.

Urethral duplication can be observed in a context of complex urogenital malformations [2]. Until a certain age of intrauterine life, the development of the urinary tract is fully integrated with that of the genital tract in both males and females. In females, the cranial part of the pelvic portion of the final urogenital sinus remains narrow, constituting the very short female urethra. The pelvic part gradually reduces in depth and is incorporated into the phallic portion, leading to the urethral and vaginal orifices which open into the vestibule. The lower and horizontal part (phallic portion) widens and forms the vestibule, limited inferiorly by the urogenital membrane which breaks during the 7th week of embryonic life (Figure 7) [7]. This closeness during embryonic development thus explains the frequent association of urinary and genital malformations. This is the case in our observation where there is an association of urethral duplication by persistence of the phallic urethra with cystic exteriorization to the vulva, the urogenital sinus, and the uterus didelphys.

Besides endoscopy, the main work-up examinations include a voiding cystography showing the main channel as well as retrograde opacification from the accessory canal [2, 5]. In our case, voiding radiographs revealing the two ureters simultaneously were not obtained, but the two-stage opacification of each urethral orifice made it possible to visualize the urethral pathways and their orifices. The plain films also revealed a pubic symphysis disjunction, which is often associated and must be systematically verified [1].

Ultrasound of the urinary tract can demonstrate the exact length of each urethra, looking for stenosis, diverticulations, and anomalies of the periurethral soft tissues as well as associated malformations such as bladder, ureteral duplication, or renal agenesis [1]. It has the advantage of being nonirradiating but it is an operator-dependent examination. In our case, it confirmed the existence of an intrapelvic bladder, which eliminated the hypothesis of a cystocele which could be mistaken for the vulvar cyst, and the existence of upper urinary tract malformations.

MRI provides a more accurate malformation assessment for the evaluation of urethral duplication by precisely demonstrating the sizes, shapes, and positions of both urethras as well as the existence of other genitourinary anomalies [1, 4]. But it is not always available or in our context.

Antenatal morphological ultrasound demonstrates once again its importance, having made it possible in our observation to demonstrate the vulvar cyst, the equinovarus foot, and the single umbilical artery. The family was quickly oriented towards a hospital structure adapted to the management of this kind of condition. An elective Caesarean section was performed and the newborn was treated early, the surgical act consisting of an excision of the cystic accessory phallic urethra with urogenital sinus septation and vaginoplasty. There were no postoperative complications.

Our patient presented with other bone abnormalities associated with the left foot equinovarus deformity; these included sacrococcygeal malformations and partial agenesis of the left tibia. These malformations can be integrated within the framework of a VACTERL and must trigger the verification of other malformations including cardiovascular and tracheoesophageal malformations.

## 4. Conclusion

Urethral duplication is a rare malformation especially in girls. The diagnosis of the anatomical form relies mainly in our context on voiding and retrograde cystography. Associated anomalies, in particular urogenital, should be systematically verified.
